# Effect of Corneal Incision Features on Corneal Endothelial Cell, Astigmatism, and Higher‐Order Aberrations After Implantable Collamer Lens Implantation

**DOI:** 10.1155/joph/8721135

**Published:** 2026-01-22

**Authors:** I-Chun Lin, Mingrui Cheng, Mingwei Li, Yinjie Jiang, Guanghan Xu, Yadi Lei, Zhiwei Mao, Rui Ning, Xun Chen, Xiaoying Wang

**Affiliations:** ^1^ Department of Ophthalmology, Fudan University Eye Ear Nose and Throat Hospital, Shanghai, China; ^2^ Key Laboratory of Myopia and Related Eye Diseases, NHC and Chinese Academy of Medical Sciences, Shanghai, China; ^3^ Shanghai Research Center of Ophthalmology and Optometry, Shanghai, China; ^4^ State Key Laboratory of Medical Neurobiology and MOE Frontiers Center for Brain Science, Fudan University, Shanghai, China, fudan.edu.cn

**Keywords:** astigmatism, clear corneal incision, endothelial cell density, higher-order aberrations, implantable collamer lens

## Abstract

**Purpose:**

This study evaluated the impact of clear corneal incision (CCI) morphology on corneal endothelial cell density (ECD), astigmatism, and higher‐order aberrations (HOAs) following implantable collamer lens (ICL) implantation.

**Design:**

Prospective study.

**Methods:**

Sixty‐five patients (65 eyes) undergoing ICL implantation were included. CCI characteristics were assessed with the AS‐OCT CASIA 2, and HOAs were measured using the Pentacam. Corneal astigmatism changes were analyzed using Aplin’s vector analysis.

**Results:**

The study divided the eyes into two groups: intact incision (29 eyes, with well‐aligned wound architecture) and defective incision (36 eyes, with misaligned incision planes or endothelial gaps). At 3 months postoperatively, corneal thickness at the incision exit (CT‐Ex) was significantly different between the groups (*p* = 0.038). The incision exit angle (Angle‐Ex) also differed significantly at 1 month (*p* = 0.023) and 3 months (*p* = 0.022). The defective incision group showed increases in incision length (IL) and the distance from the entry incision to the center (Dis‐En) between 1 and 3 months (*p* = 0.006 and *p* = 0.034, respectively). Both groups showed significant reductions in CT‐Ex, CT‐En, and Angle‐Ex at 3 months. In the defective incision group, trefoil, quadrafoil, and spherical aberrations increased significantly (*p* < 0.05) while ECD decreased significantly (*p* < 0.05).

**Conclusions:**

Shorter IL and smaller exit angle during ICL surgery increase the risk of forming defective incisions, leading to more HOAs and greater ECD loss within the 3‐month postoperative period.

**Synopsis**

• This study explores how clear corneal incision morphology affects endothelial cell density, astigmatism, and aberrations after ICL implantation, highlighting the risks associated with shorter incisions and smaller incision exit angles.

## 1. Introduction

Uncorrected refractive error is a leading cause of distance vision impairment globally, and the prevalence of myopia is steadily increasing [1]. Refractive surgery improves uncorrected visual acuity in patients with myopia. The implantable collamer lens (ICL; Staar Surgical, Nidau, Switzerland), a posterior chamber phakic intraocular lens (pIOL) approved by the U.S. Food and Drug Administration, is widely used for myopia correction. ICL implantation is reversible and preserves corneal integrity, enhancing visual acuity by correcting myopia (up to −18 D) and astigmatism (up to 6 D). Numerous studies have confirmed the safety, efficacy, and positive outcomes of ICL implantation [2–4].

ICL implantation is performed through a corneal incision that minimally affects corneal shape; therefore, this implantation has a significantly lower likelihood of inducing corneal higher‐order aberrations (HOAs). Several studies comparing HOAs between ICL implantation and corneal refractive surgeries have found that ICL surgery induces fewer HOAs than corneal refractive surgeries [5–8]. However, clear corneal incisions (CCIs) resulting in surgically induced astigmatism (SIA) can affect the corneal optical quality [9, 10]. Studies have demonstrated that corneal morphology correlates with CCI characteristics, which in turn affect corneal recovery and may lead to HOAs following cataract surgery [11]. Compared with cataract surgery, ICL surgery involves a shorter operative time, and the cornea is not exposed to excessive energy. However, the CCI is larger in ICL surgery (3.0 mm) than in cataract surgery (2.2 mm) [12].

The correlation between the characteristics and morphology of CCIs in ICL surgery and their influence on HOAs is currently unknown. Therefore, this study aimed to analyze the characteristics of CCI morphology using specific parameters and evaluate their impact on corneal endothelial cell density (ECD), extent of astigmatism, and HOAs.

## 2. Methods

### 2.1. Patients

This prospective study included patients who underwent ICL V4c implantation at the Eye, Ear, Nose, and Throat Hospitals of Fudan University (Shanghai, China). Sixty‐five eyes of 65 patients (9 males, 56 females; mean age, 27.78 ± 5.63 years [19–46]) were included. One eye per patient was randomly selected. Two groups, the intact incision group and the defective incision group, were divided based on the morphology of CCIs measured by anterior segment optical coherence tomography (AS‐OCT). All patients provided written informed consent and received a detailed explanation of the procedure before implantation. This study adhered to the principles of the Declaration of Helsinki, and the Fudan University Eye, Ear, Nose, and Throat Hospital Ethics Committee approved the study and all methods. The names of the patients were blinded.

We followed the methods of Lin et al. and Chen et al. [13, 14]. The inclusion criteria for the study were as follows: (1) participants aged 18 years or older; (2) individuals with a stable refractive error (change of less than 0.50 D per year) for at least 2 years; (3) those with an anterior chamber depth (ACD) of 2.8 mm or greater; and (4) individuals with an ECD of 2000 cells/mm^2^ or higher. Patients were excluded if they had other ocular diseases, a history of ocular surgery or trauma, systemic diseases affecting the eyes, or serious mental illnesses.

### 2.2. Main Refractive and Biometric Measures

All patients underwent comprehensive ophthalmic evaluations both before and after surgery, which included the following assessments: (1) uncorrected distance visual acuity (UDVA) and corrected distance visual acuity (CDVA); (2) manifest and cycloplegic refraction; (3) slit‐lamp biomicroscopy and fundoscopic examinations; (4) intraocular pressure (IOP, Tonemeter X‐10, Canon, Japan); (5) corneal topography and central corneal thickness (Pentacam HR, Type 70,900; Oculus Optikgeräte GmbH, Wetzlar, Germany); (6) corneal astigmatism (Pentacam); (7) horizontal corneal diameter (white‐to‐white [WTW] (IOL Master 500, ZEISS, Germany); (8) axial length (IOL Master); (9) ACD (Pentacam); (10) ECD (SP‐3000P, Topcon Corporation, Tokyo, Japan); (11) optical coherence tomography (OCT, Optovue, Florida, USA); and (12) ultrasound biomicroscopy (UBM, Quantel Medical, Clermont‐Ferrand, France). This comprehensive evaluation ensured a thorough assessment of each patient’s ocular health and surgical candidacy.

### 2.3. HOAs and Astigmatism Vector Analysis

HOAs were measured preoperatively and at 1 and 3 months postoperatively using the Pentacam. The root mean square (RMS) of the total higher‐order aberrations (tHOAs) and individual Zernike terms—trefoil (RMS of Z[3, −3] and Z[3, 3]), coma (RMS of Z[3, −1] and Z[3, 1]), quadrafoil (RMS of Z[4, −4] and Z[4, 4]), and spherical aberration (RMS of Z[4, 0])—was calculated using Zernike polynomial coefficients at the central 6‐mm diameter zones on the total, anterior, and posterior corneal surfaces. Aplin’s vector analysis [15] was used to assess changes in corneal astigmatism induced by the incision. SIA, target‐induced astigmatism (TIA), difference vector (DV), and correction index (CI) were calculated for both groups.

### 2.4. CCI Assessments

CCI was assessed using swept‐source AS‐OCT (SS‐1000 CASIA 2, Tomey Corp., Aichi, Japan) at 1 and 3 months postoperatively. The cross‐sectional CCI images were categorized as follows: (a) intact incision; (b) wound misalignment; and (c) endothelial gap (Figure 1). Incisions with misalignment or an endothelial gap were categorized as defective. The incision parameters were measured using the package caliper tool of the Tomey Measurement software (Tomey Corp.). The CCI was measured according to the methods described by He et al. [11] Measurements included (1) incision length (IL); (2) the angle between the incision and the corneal epithelium (incision entry angle, Angle‐En); (3) the angle between the incision and the corneal endothelium (incision exit angle, Angle‐Ex); (4) corneal thickness (CT) at the incision entry (CT‐En); (5) CT at the incision exit (CT‐Ex); (6) the distance from the incision entry to the central cornea (incision entry distance, Dis‐En); and (7) the distance from the incision exit to the central cornea (incision exit distance, Dis‐Ex).

Figure 1AS‐OCT images. Representative AS‐OCT images of 3.0‐mm clear corneal incisions. (a) Intact incision: well‐aligned wound architecture; (b) Defective incision: misaligned incision planes or endothelial gaps. AS‐OCT: anterior segment optical coherence tomography.

**Figure:**
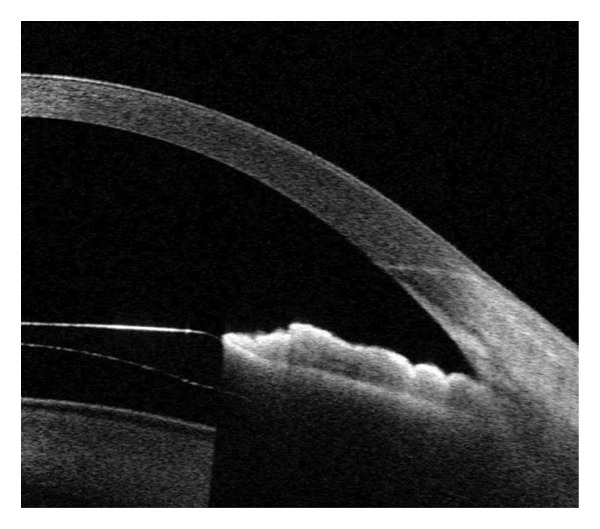
(a)

**Figure:**
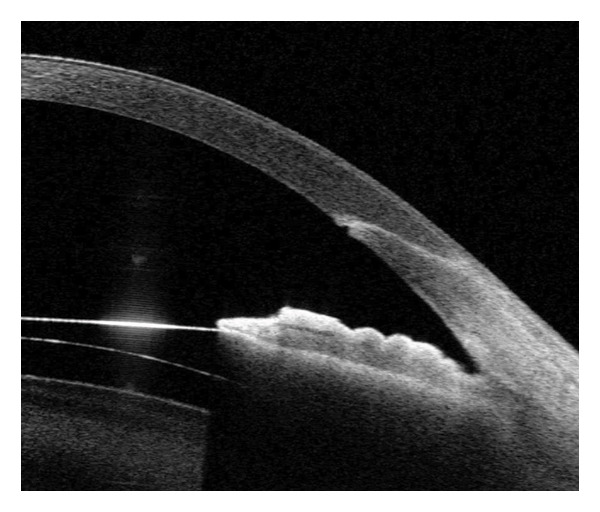
(b)

### 2.5. Lens Power and Size Calculation

The power calculation for the ICL V4c was performed using a modified vertex formula based on the preoperative refractive parameters provided by the manufacturer. The size of the implanted ICL V4c was determined by measuring the WTW horizontal corneal diameter and ACD. Additionally, the toric ICL V4c was selected for eyes with astigmatism greater than 0.5 D for those that can achieve an improvement of two or more lines in CDVA following astigmatism correction.

### 2.6. Surgical Procedure

ICL V4c implantation followed a standardized single‐step approach as outlined in previous research [6]. All surgeries were performed by an experienced surgeon (Xiaoying Wang). Preoperatively, patients were treated with 0.5% ofloxacin eye drops (Santen, Japan) four times daily for 3 days. On the day of surgery, pupils were dilated, and 0.4% oxybuprocaine was administered for local anesthesia. A 3‐mm corneal incision was made, generally along the horizontal meridian. For patients with high preoperative astigmatism (> 1.00 D), the incision was placed along the steep meridian (vertical/superior) to correct astigmatism, and 1% sodium hyaluronate was injected into the anterior chamber as a viscoelastic agent. The ICL V4c was implanted into the posterior chamber using an injector cartridge and carefully positioned to ensure proper vaulting. The viscoelastic agent was subsequently fully removed with a balanced salt solution. Postoperative care included 1.0% prednisolone acetate (four times daily for 4 days), 0.5% levofloxacin (four times daily for 7 days), pranoprofen (four times daily for 14 days), and artificial tears (four times daily for 1 month).

### 2.7. Statistical Analysis

SPSS (Version 26.0; SPSS Inc., IBM, USA) was used for data analyses. The results are expressed as the mean ± standard deviation. The Kolmogorov–Smirnov test was used to confirm the normality of the statistical analyses. Student’s *t*‐test was performed for normally distributed variables, whereas the Wilcoxon rank‐sum test was applied for non‐normally distributed variables. A paired *t*‐test or Wilcoxon signed‐rank test was used to compare parameters between two groups preoperatively and at 1 and 3 months postoperatively. One‐way analysis of variance (ANOVA) was applied to compare the HOAs preoperatively and at 1 and 3 months postoperatively. Pearson’s correlation coefficient was used to analyze the correlations between categorical variables. Two‐tailed hypothesis testing was performed, and statistical significance was set at *p* < 0.05.

## 3. Results

### 3.1. Patient Profiles

Sixty‐five eyes of 65 patients were followed up at 1 and 3 months after ICL implantation. Table 1 shows the preoperative demographic and clinical characteristics of the included patients and parameters of the eyes. The mean spherical diopter was 0.16 ± 0.18 D in the intact incision group and −0.03 ± 0.55 D in the defective incision group at the 1‐month follow‐up (*p* = 0.063), 0.06 ± 0.25 D and −0.03 ± 0.54 D (*p* = 0.773) at the 3‐month follow‐up, respectively. The mean cylinder diopter was −0.30 ± 0.26 D in the intact incision group and −0.38 ± 0.37 D in the defective incision group at the 1‐month follow‐up (*p* = 0.906), −0.19 ± 0.23 D and −0.30 ± 0.28 D (*p* = 0.091) at the 3‐month follow‐up, respectively.

**Table 1:** Patient profiles.

	Intact incision (*n* = 29)	Defective incision (*n* = 36)	*p*
	Mean ± SD	Mean ± SD	
Age (years)	27.79 ± 5.15	27.78 ± 6.06	0.991
Gender (male/female)	5/24	4/32	
Axial Length (mm)	26.65 ± 1.15	26.94 ± 1.06	0.300
Pre‐Sphere (D)	−7.99 ± 1.54	−7.86 ± 1.76	0.917
Pre‐Cylinder (D)	−1.04 ± 0.61	−0.97 ± 0.57	0.663
Pre‐SE (D)	−8.22 ± 2.22	−8.34 ± 1.84	0.973
Pre‐CDVA (logMAR)	−0.00 ± 0.04	−0.01 ± 0.07	0.452

Abbreviations: D, diopter; SD, standard deviation; SE, spherical equivalent.

### 3.2. Safety and Efficacy

The mean safety indices (postoperative CDVA/preoperative CDVA) were 1.30 ± 0.22 for the intact incision group and 1.25 ± 0.19 for the defective incision group (*p* = 0.305) at 1 month after surgery, 1.36 ± 0.20 and 1.33 ± 0.20 (*p* = 0.529) at 3 months after surgery, respectively. None of the eyes in either group lost one or more CDVA lines (Figures 2(a), 2(b)). The mean logMAR CDVA values were −0.00 ± 0.04 in the intact incision group and −0.01 ± 0.07 (*p* = 0.452) in the defective incision group at baseline, −0.13 ± 0.07 and −0.12 ± 0.07 (*p* = 0.487) at the 1‐month follow‐up, −0.15 ± 0.06 and −0.15 ± 0.06 (*p* = 0.941) at the 3‐month follow‐up, respectively.

Figure 2Refractive outcomes at 1 and 3 months after implantable collamer lens (ICL) implantation in the intact incision group and defective incision group. (a) Changes in Snellen lines of corrected distance visual acuity (CDVA) at the 1‐month follow‐up in the intact and defective incision groups; (b) changes in Snellen lines of CDVA at the 3‐month follow‐up in the intact and defective incision groups; (c) changes in Snellen lines of uncorrected distance visual acuity (UDVA) at the 1‐month follow‐up in the intact and defective incision groups; and (d) changes in Snellen lines of UDVA at the 3‐month follow‐up in the intact and defective incision groups.

**Figure:**
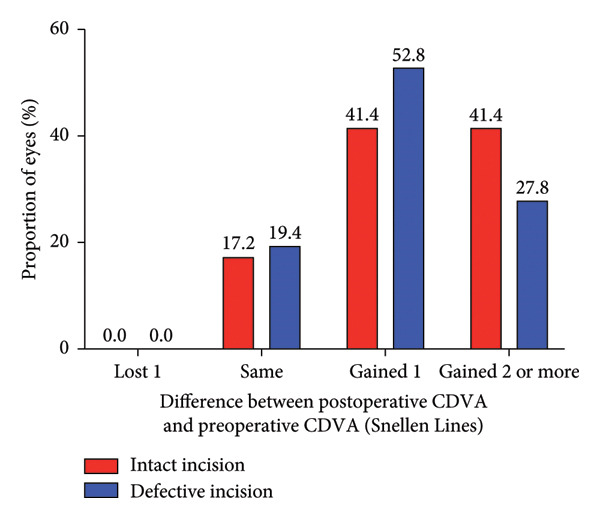
(a)

**Figure:**
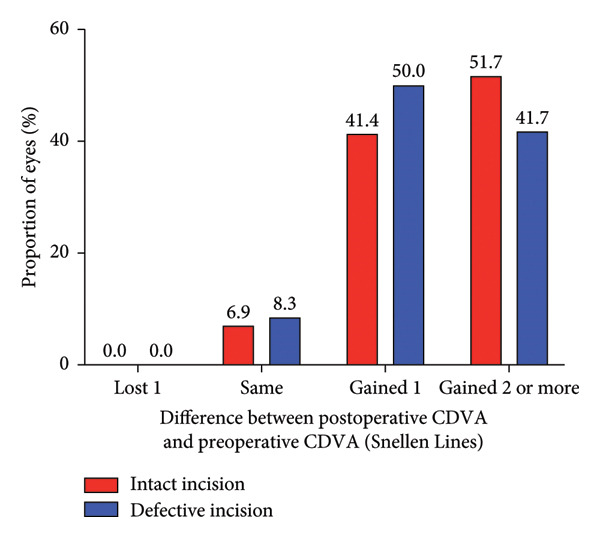
(b)

**Figure:**
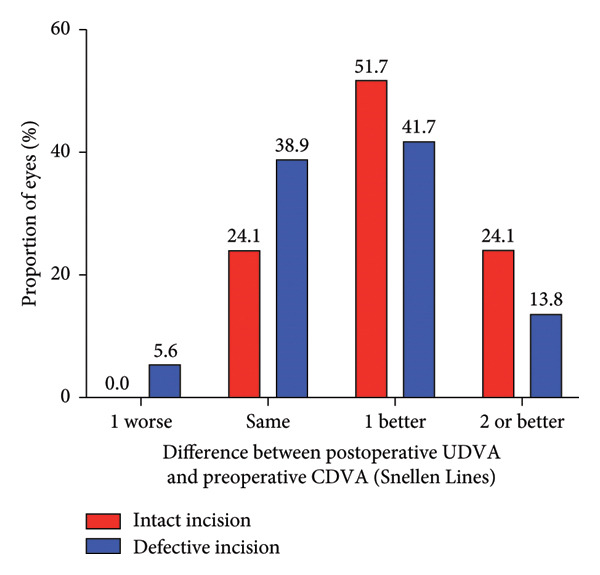
(c)

**Figure:**
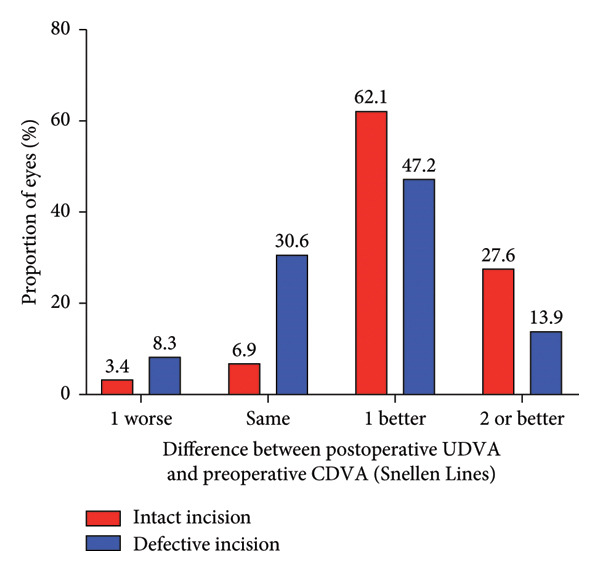
(d)

The mean efficacy indices (postoperative UDVA/preoperative CDVA) were 1.24 ± 0.21 for the intact incision group and 1.14 ± 0.18 for the defective incision group (*p* = 0.067) at 1 month after surgery, 1.27 ± 0.20 and 1.16 ± 0.19 (*p* = 0.020) at 3 months after surgery, respectively (Figures 2(c), 2(d)). The mean logMAR UDVA values were −0.11 ± 0.07 in the intact incision group and −0.08 ± 0.08 in the defective incision group at the 1‐month follow‐up (*p* = 0.118) and −0.12 ± 0.06 and −0.08 ± 0.09 at the 3‐month follow‐up (*p* = 0.062), respectively. All eyes (29 of 29) in the intact incision group and 94% (34 of 36) of the eyes in the defective incision group demonstrated a UDVA equal to or better than that of the preoperative CDVA at the 1‐month follow‐up, 97% (28 of 29) and 92% (33 of 36) at the 3‐month follow‐up, respectively. None of the eyes in either group lost more than one line of UDVA postoperatively compared with their preoperative CDVA (Figures 3(a), 3(b)).

Figure 3Refractive outcomes at 3 months after implantable collamer lens (ICL) implantation. (a) Attempted versus achieved spherical equivalent (SE) refraction at the 3‐month follow‐up in the intact incision group; (b) attempted versus achieved SE refraction at the 3‐month follow‐up in the defective incision group; (c) stability of SE refraction assessed at the preoperative, 1‐month, and 3‐month follow‐ups in the intact incision group; and (d) stability of SE refraction assessed at the preoperative, 1‐month, and 3‐month follow‐ups in the defective incision group. D: diopters; Pre‐op: preoperative.

**Figure:**
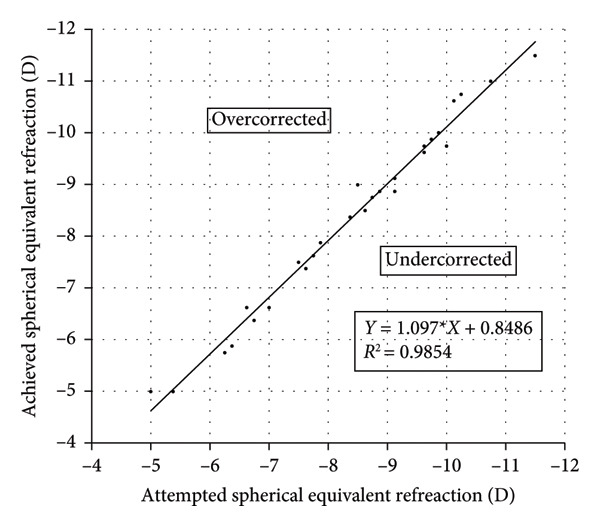
(a)

**Figure:**
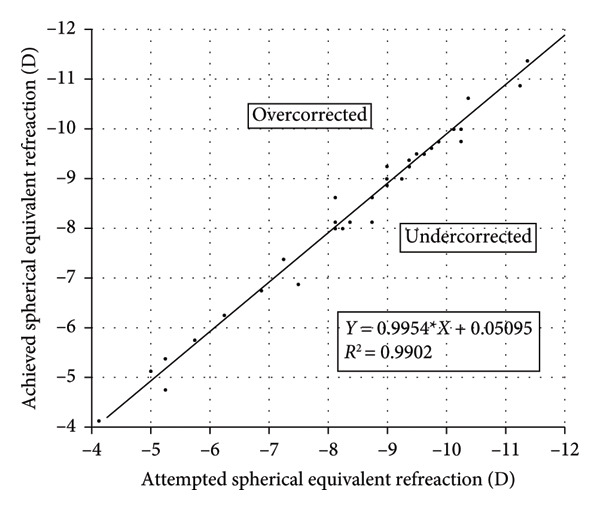
(b)

**Figure:**
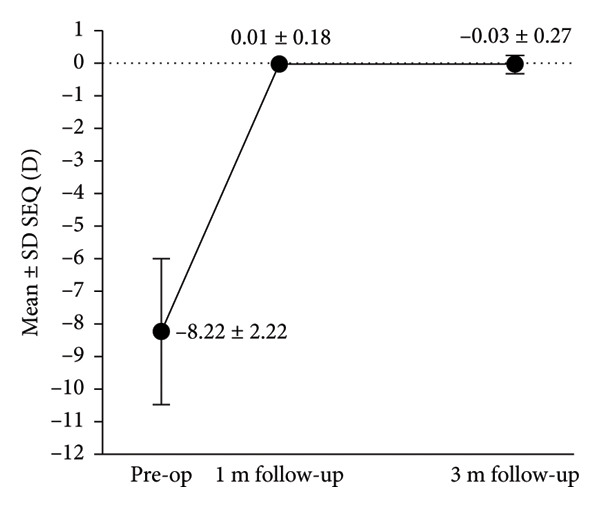
(c)

**Figure:**
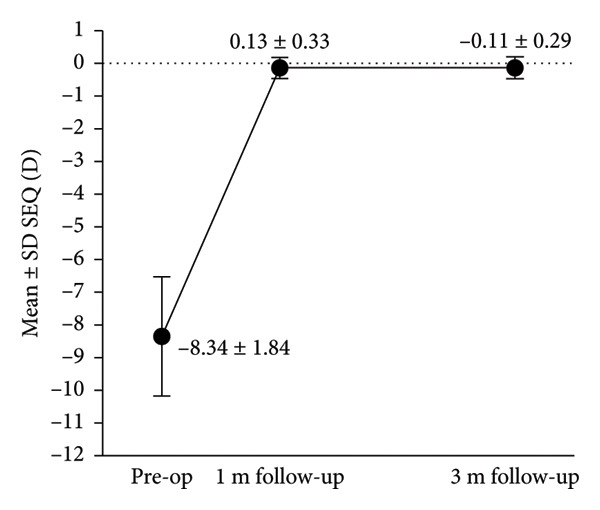
(d)

### 3.3. Predictability and Stability

A scatter plot of the attempted versus achieved spherical equivalent (SE) at the 3‐month follow‐up in the intact and defective incision groups is shown in Figures 3(a), 3(b). At the 3‐month follow‐up, a comparison of the preoperative SE and the target refractive power revealed that all eyes (29/29) were within ±0.50 D in the intact incision group, whereas 91.6% (33/36) of the eyes were within ±0.50 D and all (36/36) were within ±0.75 D in the defective incision group.

The mean SE decreased from −8.22 ± 2.22 D preoperatively to 0.01 ± 0.18 D at the 1‐month follow‐up and −0.03 ± 0.27 D at the 3‐month follow‐up postoperatively in the intact incision group and from −8.34 ± 1.84 D to −0.13 ± 0.33 D and −0.11 ± 0.29 D in the defective incision group, respectively (Figures 3(c), 3(d)).

### 3.4. CCI Feature Assessments

Table 2 presents the assessments of CCI features at 1 and 3 months postoperatively in the intact and defective incision groups. Three months postoperatively, the results showed a significant difference in CT‐Ex between the two groups (*p* = 0.038). Additionally, Angle‐Ex was significantly different between the groups at 1 month (*p* = 0.023) and 3 months (*p* = 0.022) postoperatively. In the defective incision group, the IL and Dis‐En were both significantly larger at 3 months than at 1 month postoperatively (*p* = 0.006 and *p* = 0.034, respectively). In both the intact and defective incision groups, CT‐Ex (*p* < 0.001 and *p* < 0.001, respectively), CT‐En (*p* = 0.002 and *p* < 0.001, respectively), and Angle‐Ex (*p* = 0.002 and *p* < 0.001, respectively) showed significant reductions at 3 months postoperatively compared with those at 1 month postoperatively.

**Table 2:** Clear corneal incision feature assessments at 1‐ and 3‐month follow‐ups.

	**1-month follow-up**			**3-month follow-up**				
	**Intact incision (*n* = 29)**	**Defective incision (*n* = 36)**		**Intact incision (*n* = 29)**	**Defective incision (*n* = 36)**			
	**Mean ± SD**	**Mean ± SD**	**p**_1_	**Mean ± SD**	**Mean ± SD**	**p**_2_	**p**_3_	**p**_4_

IL	1.42 ± 0.20	1.39 ± 0.20	0.501	1.44 ± 0.22	1.42 ± 0.22	0.725	0.173	0.006^∗^
Dis‐Ex (mm)	4.20 ± 0.58	4.07 ± 0.48	0.348	4.20 ± 0.60	4.07 ± 0.53	0.356	0.819	0.930
Dis‐En (mm)	5.58 ± 0.71	5.44 ± 0.58	0.394	5.60 ± 0.70	5.49 ± 0.62	0.507	0.390	0.034^∗^
CT‐Ex (mm)	0.72 ± 0.04	0.71 ± 0.04	0.181	0.70 ± 0.04	0.68 ± 0.04	0.093	< 0.001^∗∗^	< 0.001^∗∗^
CT‐En (mm)	0.85 ± 0.09	0.82 ± 0.08	0.128	0.83 ± 0.09	0.79 ± 0.08	0.038^∗^	0.002^∗^	< 0.001^∗∗^
Angle‐Ex (°)	40.97 ± 5.85	38.01 ± 4.35	0.023^∗^	38.31 ± 5.84	35.44 ± 3.96	0.022^∗^	0.002^∗^	< 0.001^∗∗^
Angle‐En (°)	33.14 ± 15.79	33.61 ± 13.31	0.898	32.78 ± 15.70	32.29 ± 12.76	0.889	0.567	0.022^∗^

*Note:* *p*_1_: differences between intact and defective incision at 1‐month follow‐up, *p*_2_: differences between intact and defective incision at 3‐month follow‐up; *p*_3_: differences in intact incision between 1‐ and 3‐month follow‐ups; *p*_4_: differences in defective incision between 1‐ and 3‐month follow‐ups; CT‐En = corneal thickness at incision entry, CT‐Ex = corneal thickness at incision exit, Dis‐En = the distance from incision entry to central cornea, Dis‐Ex = the distance from incision exit to central cornea, IL = incision length.

Abbreviation: SD, standard deviation.

^∗^ *p* < 0.05.

^∗∗^ *p* < 0.001.

### 3.5. Corneal Astigmatism Change Analysis

Figures 4(a), 4(b) illustrate the changes in corneal astigmatism—including SIA, TIA, DV, and CI—at 3 months postoperatively based on Aplin’s vector analysis in the intact and defective incision groups. Postoperatively, no significant differences between the two groups were observed in any of these parameters (*p* > 0.05).

Figure 4Distribution of corneal astigmatism changes. Changes in surgically induced astigmatism (SIA), target‐induced astigmatism (TIA), difference vector (DV), and correction index (CI) at 3 months postoperatively following implantable collamer lens (ICL) implantation. (a) Distribution of corneal astigmatism changes 3 months after surgery in the intact incision group; (b) distribution of corneal astigmatism changes 3 months after surgery in the defective incision group.

**Figure:**
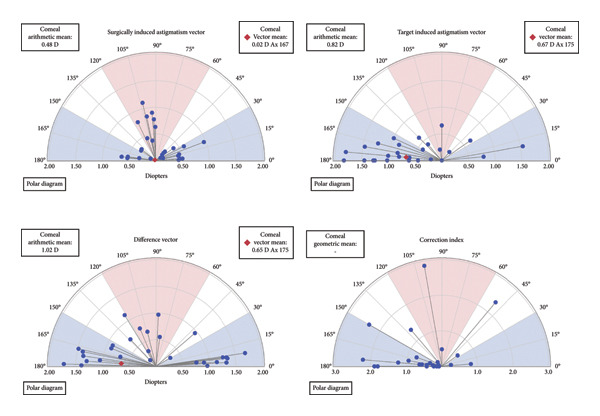
(a)

**Figure:**
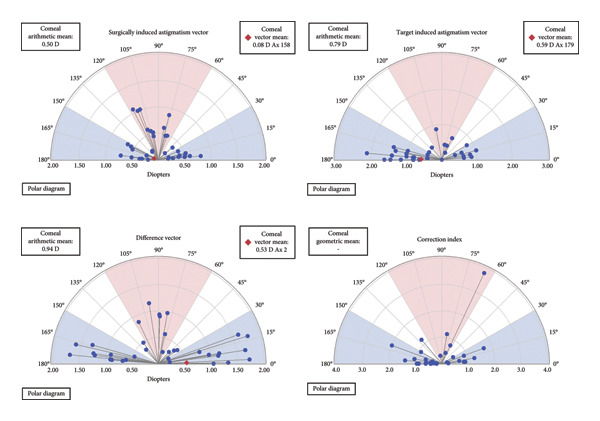
(b)

### 3.6. Corneal HOA Analysis

For total corneal HOAs, quadrafoil aberrations significantly increased 1 month after surgery in the intact incision group (*p* = 0.011). In the defective incision group, trefoil, quadrafoil, and spherical aberrations significantly increased at 1 month postoperatively (*p* = 0.012, *p* < 0.001, and *p* = 0.029, respectively). Additionally, in the defective incision group, quadrafoil and spherical aberrations remained significantly elevated 3 months after surgery (*p* = 0.005 and *p* = 0.015, respectively). There was no significant difference in HOAs between the two groups preoperatively and postoperatively (Figure 5(a)).

Figure 5Higher‐order aberrations (HOAs). HOAs at the 6‐mm zone for the intact and defective incision groups preoperatively and at 1 and 3 months after ICL implantation. (a) Total corneal HOAs; (b) anterior corneal surface HOAs; and (c) posterior corneal surface HOAs.

**Figure:**
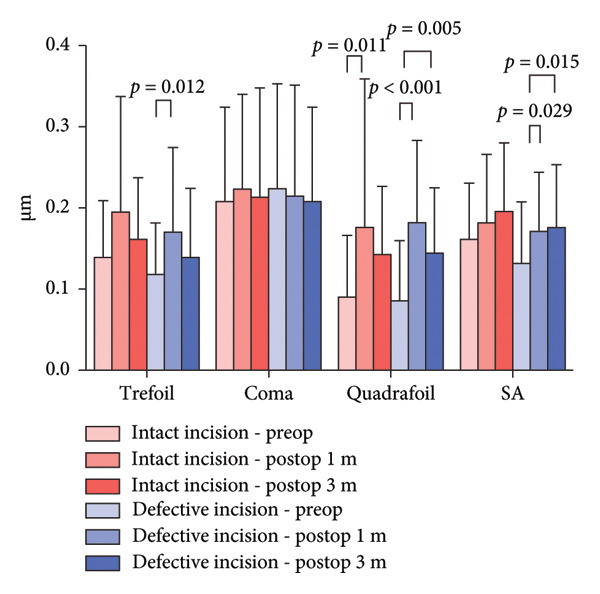
(a)

**Figure:**
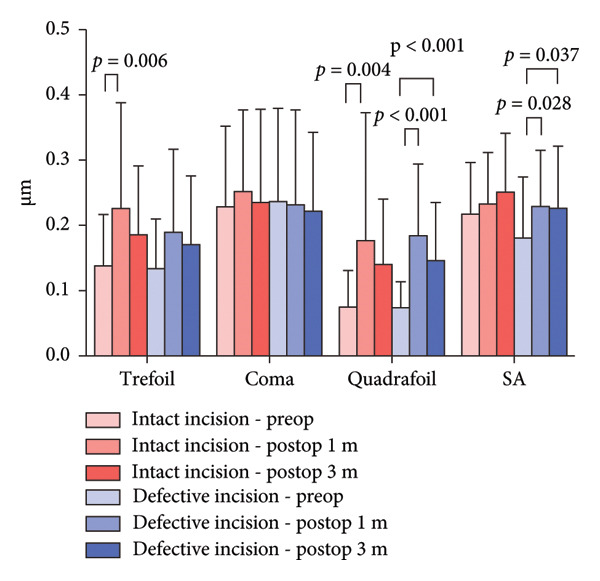
(b)

**Figure:**
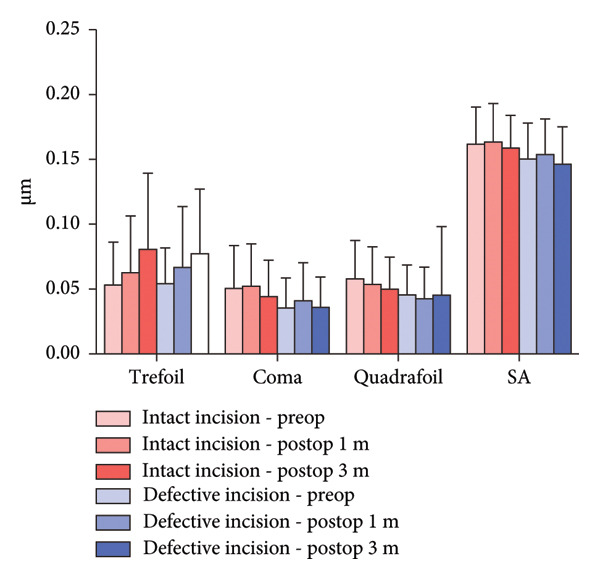
(c)

Regarding anterior corneal HOAs, trefoil and quadrafoil aberrations significantly increased at 1 month postoperatively in the intact incision group (*p* = 0.006 and *p* = 0.004, respectively). In the defective incision group, quadrafoil and spherical aberrations significantly increased at 1 month postoperatively (*p* < 0.001 and *p* = 0.028, respectively) and at 3 months postoperatively (*p* < 0.001 and *p* = 0.037, respectively). There was no significant difference in HOAs between the two groups preoperatively and postoperatively (Figure 5(b)).

Regarding posterior corneal HOAs, there was no significant difference between the preoperative and postoperative HOAs in the intact and defective incision groups. Additionally, there was no significant difference in HOAs between the two groups preoperatively and postoperatively (Figure 5(c)).

### 3.7. Central Corneal ECD

Table 3 shows the comparison of ECD between the two groups preoperatively and 3 months postoperatively. In the defective incision group, ECD at 3 months after ICL implantation was significantly lower than the preoperative value (*p* < 0.05). In contrast, the intact incision group showed no significant differences in ECD between the preoperative and 3‐month postoperative values (*p* > 0.05). Additionally, no significant differences in ECD were observed between the intact and defective incision groups at any of the preoperative and 3‐month follow‐up assessments (*p* > 0.05).

**Table 3:** Comparison of corneal endothelial cell density between the two groups at preoperative, 1‐month, and 3‐month follow‐ups.

	Intact incision (*n* = 29)	Defective incision (*n* = 36)	*p*_1_
	Mean ± SD	Mean ± SD	
Preoperative (cells/mm^2^)	2789 ± 231	2854 ± 247	0.293
3‐month follow‐up (cells/mm^2^)	2779 ± 224	2732 ± 202	0.408
*p*_2_	0.627	0.002^∗^	

*Note:* *p*_1_: difference between intact and defective incision groups at preoperative and 3‐month follow‐ups; *p*_2_: difference between preoperative and 3‐month follow‐up in the intact and defective incision groups, respectively.

Abbreviation: SD, standard deviation.

^∗^ *p* < 0.05.

## 4. Discussion

ICL is a surgical procedure designed to correct myopia by implanting a pIOL through a 3‐mm CCI. Although the procedure does not involve corneal ablation, a 3‐mm CCI may induce SIA and alter the corneal architecture. These changes may affect corneal curvature and, consequently, postoperative visual quality. This study used OCT to classify postoperative incision closure into two categories—intact incision and defective incision—and grouped the eyes accordingly. The intact incision group included patients with well‐aligned incisions, while the defective incision group included patients with misaligned incisions and endothelial gaps. The characteristics of the incisions and corneal HOAs were compared between the two groups.

Regarding CCI features, the Angle‐Ex was significantly larger in the intact incision group than in the defective incision group at 1 and 3 months postoperatively. The construction of the CCI, including the angle of incision, plays a crucial role in preventing leaks and ensuring postoperative stability. A previous study reported that the stable incision angle range for single‐planed incisions was between 30° and 40° in ex vivo human eyes, and a two‐planed incision can effectively expand the stable range [16]. All patients included in the present study underwent a two‐plane incision. Our study showed that a larger Angle‐Ex resulted in better self‐sealing properties and prevented endothelial gaping and wound misalignment. We observed a decrease in CT‐Ex and CT‐En as the postoperative corneal incisional edema gradually decreased 1–3 months after surgery. As this edema decreased, the outward extension of the corneal incision exit resulted in increased IL and Dis‐Ex, with corresponding decreases in Angle‐En and Angle‐Ex, particularly in the defective incision group. We assumed that the defective incisions caused more corneal edema in the short term after surgery than the intact incisions. As the postoperative edema decreased, the CCI features changed more significantly in the defective incision group than in the intact incision group.

Regarding the HOAs in the central 6‐mm diameter zones, both the intact and defective incision groups showed a significant increase in trefoil and quadrafoil aberrations in the whole and anterior corneal surfaces. However, only the defective incision group demonstrated an increase in spherical aberrations after surgery in both the whole and anterior corneal surfaces.

No significant differences were observed pre‐ or postoperatively in either group in the posterior corneal surface. Furthermore, there was no significant difference in the corneal HOAs between the two groups.

HOAs were measured over a 6.0 mm central corneal zone. Because the incision was located near the corneal periphery, this measurement range may have limited the detection of peripheral HOA changes, potentially explaining the lack of significant differences between groups.

Despite the defective incision causing more corneal edema and increased spherical aberrations postoperatively than the intact incision, we found no differences in visual acuity, refractive diopters, or incision‐induced astigmatism between the two groups at 1 and 3 months postoperatively. We speculate that this may be explained by the shorter operative time and comparatively less instrument contact involved in ICL surgery. Although the CCI characteristics had flaws, such as endothelial gaps and misaligned incisions, they did not significantly impact the overall visual quality or precision of the target astigmatism intended to be induced.

The morphological characteristics of the CCI appear to influence the central endothelial cell loss after surgery [17]. In the defective incision group, ECD showed a 4.27% decrease from preoperative levels at 3 months after ICL implantation. In contrast, the intact incision group showed a 0.36% reduction over 3 months. Kisiel and Gurumurthy indicated that acute surgical trauma is the primary contributor to ECD loss at 3 months postsurgery [18]. Our findings demonstrated that intact incisions resulted in reduced ECD loss within 3 months postoperatively.

This study has some limitations that must be considered when interpreting and applying the results. First, the sample size was relatively small, which may have affected the statistical significance of the results. Additionally, the follow‐up period was only extended to 3 months, meaning that long‐term changes in CCI features and HOAs were not assessed.

## 5. Conclusion

In conclusion, our results showed that a relatively short IL of the CCI and a smaller Angle‐Ex in ICL implantation increased the risk of developing a defective incision. These defects, including CCI misalignments and endothelial gaps, can introduce additional HOAs, such as quadrafoil and spherical aberrations, potentially affecting postoperative visual outcomes, and contribute to greater ECD loss within the 3‐month postoperative period.

## Ethics Statement

This study adhered to the tenets of the Declaration of Helsinki and was approved by the Ethical Committee Review Board of Fudan University Eye and ENT Hospital (2021018).

## Consent

Written informed consent was obtained from the patient for publication of this study.

## Disclosure

All authors read and approved the final manuscript.

## Conflicts of Interest

The authors declare no conflicts of interest.

## Author Contributions

Conception or design of the work, the acquisition, and analysis or interpretation of data for the work: I‐Chun Lin, Mingrui Cheng, Mingwei Li, Yinjie Jiang, Guanghan Xu, Yadi Lei, Zhiwei Mao, Rui Ning, Xun Chen, and Xiaoying Wang; drafting the work or revising it critically for important intellectual content: I‐Chun Lin and Mingrui Cheng; final approval of the version to be published: Xun Chen, Xiaoying Wang; agreement to be accountable for all aspects of the work in ensuring that questions related to the accuracy or integrity of any part of the work are appropriately investigated and resolved: all authors. I‐Chun Lin and Mingrui Cheng contributed equally and are considered co‐first authors.

## Funding

This study was supported by National Natural Science Foundation of China (Grant No. 82171095). Project of Shanghai Science and Technology (Grant No. 23XD1400500). Project of Shanghai Shenkang Hospital Development Center (Grant No. SHDC12024148).

## Data Availability

Data and materials are available upon request from the corresponding author at chenxun19910923@163.com or doctxiaoyingwang@163.com.
